# Effect of surfactant on *Pseudomonas aeruginosa* colonization of polymer microparticles and flat films†

**DOI:** 10.1039/c8ra01491d

**Published:** 2018-04-24

**Authors:** Amanda Hüsler, Simon Haas, Luke Parry, Manuel Romero, Takasi Nisisako, Paul Williams, Ricky D. Wildman, Morgan R. Alexander

**Affiliations:** Advanced Materials and Healthcare Technologies Division, School of Pharmacy, University of Nottingham Nottingham NG7 2RD UK morgan.alexander@nottingham.ac.uk; Centre for Additive Manufacturing, Faculty of Engineering, University of Nottingham Nottingham NG7 2RD UK; Institute of Innovative Research, Tokyo Institute of Technology Yokohama Japan; Centre for Biomolecular Sciences, School of Life Sciences, University of Nottingham Nottingham NG7 2RD UK

## Abstract

Micro- and nanoparticles are of great interest because of their potential for trafficking into the body for applications such as low-fouling coatings on medical devices, drug delivery in pharmaceutics and cell carriers in regenerative medicine strategies. Particle production often relies on the use of surfactants to promote stable droplet formation. However, the presence of residual surfactant has been shown to complicate the surface chemistry and resultant properties. When forming particles from polymerizable monomer droplets, these polymeric surfactant chains can become physically entangled in the particle surface. Due to the key role of the outermost layers of the surface in biomaterial interactions, the surface chemistry and its influence on cells needs to be characterized. This is the first study to assess surfactant retention on microfluidic produced particles and its effect on bacterial attachment; surfactant contaminated microparticles are compared with flat films which are surfactant-free. Polymeric microparticles with an average diameter of 76 ± 1.7 μm were produced by using a T-junction microfluidic system to form monomer droplets which were subsequently photopolymerized. Acrylate based monomer solutions were found to require 2 wt% PVA to stabilize droplet formation. ToF-SIMS was employed to assess the surface chemistry revealing the presence of PVA in a discontinuous layer on the surface of microparticles which was reduced but not removed by solvent washing. The effect of PVA on bacterial (*Pseudomonas aeruginosa*) attachment was quantified and showed reduction as a function of the amount of PVA retained at the surface. The insights gained in this study help define the structure–function relationships of the particulate biomaterial architecture, supporting materials design with biofilm control.

## Abstract 2

The attachment of *Pseudomonas aeruginosa* on microfluidic produced particles was shown to reduce as a function of PVA concentration retained at the surface, enabling novel structure–function relationships of biomaterial architecture.

## Graphical abstract

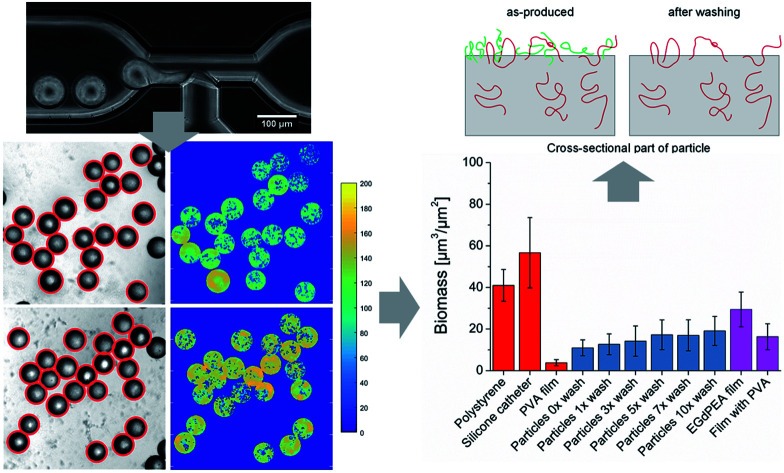

## Introduction

Over the last decade, materials discovery using high throughput screening has been applied to identify novel materials with unique properties that can be tailored for specific applications, such as maintaining stem cell pluripotency for tissue engineering applications or reducing bacterial attachment and biofilm formation for medical devices.^1–3^ Polymer microarrays allow screening of many hundreds of materials in parallel to identify a desired biological response with the ability to discover novel materials without the need to fully understand the cell–material interactions.^4^ However, this approach has only been applied to flat, or two-dimensional (2D) materials, and it is well recognized that cells can behave differently on three-dimensional (3D) or topographically patterned structures, for example by contact guidance.^5^ Consequently, the materials discovery procedures need to be expanded into the third dimension; from flat microarrays to spheres, possibly the simplest 3D shape. Particles have wide applicability for use in a range of areas including drug delivery,^6,7^ tissue engineering,^8,9^ diagnostics^10^ and photonics.^11^ They can be produced by many techniques, with the most commonly used for polymers being precipitation,^12^ emulsion polymerization^13^ and most recently, microfluidic based methods.^14,15^ Droplet formation in microfluidic devices is capable of producing highly uniform droplets ranging from the nano- to the microscale from a variety of materials.^16,17^ Particle control has been demonstrated by Kim *et al.* to be feasible with microfluidics, ranging from control of shape to compartmentalization of different components.^14^ This technique has been shown to be suitable for manufacturing large quantities of particles,^18^ and has the potential to generate particles of diverse compositions when coupled with automated fluid handling and a library of UV polymerizable monomers. A drawback that is shared among many droplet generation methods is that surfactants are often required in order to stabilize the fluid–fluid interfaces of the droplets.^19,20^ Salman *et al.* have recently reported that poly(vinyl alcohol-*co*-vinyl acetate) (PVA) and a mixture of PVA with a biosurfactant reduces the formation of *Staphylococcus aureus* and *Pseudomonas aeruginosa* biofilms by up to 98% on glass and plastic plates, depending on the molecular weight of PVA used.^21^

Novel materials that can control bacterial attachment and biofilm formation are in demand to reduce device-centered infections in the fight against antimicrobial resistance (AMR).^22^ Moreover, the adhesion of marine fouling organisms to surfaces such as ships' hulls poses a worldwide problem and is subject to on-going research.^23,24^ Microorganisms attach to surfaces and form biofilms *i.e.* populations of microbial cells enmeshed in a matrix consisting of extracellular polymers such as polysaccharides, proteins and nucleic acids. In such bacterial communities, bacteria are up to 1000 times more tolerant to antimicrobials and host defenses than individual planktonic bacterial cells.^25^ Addressing this at the earliest possible stage ideally requires that surfaces are engineered in such a way that they can prevent bacterial attachment and subsequent biofilm formation.

In this paper, the production of monodisperse microparticles with an average diameter of 76 ± 1.7 μm was demonstrated using a simple microfluidic T-junction configuration by photopolymerizing acrylate based monomer solutions. First, we show the need for a surfactant, in this case 2 wt% PVA, for successful droplet formation. Then, the surface chemistry of the fabricated particles was assessed using Time-of-Flight secondary ion mass spectrometry (ToF-SIMS). The effect of PVA on the attachment of *P. aeruginosa* was investigated on acrylate-based microparticles using washing cycles to control the amount of PVA.

## Results and discussion

### Formation of microparticles in a T-junction

**Fig. 1:**
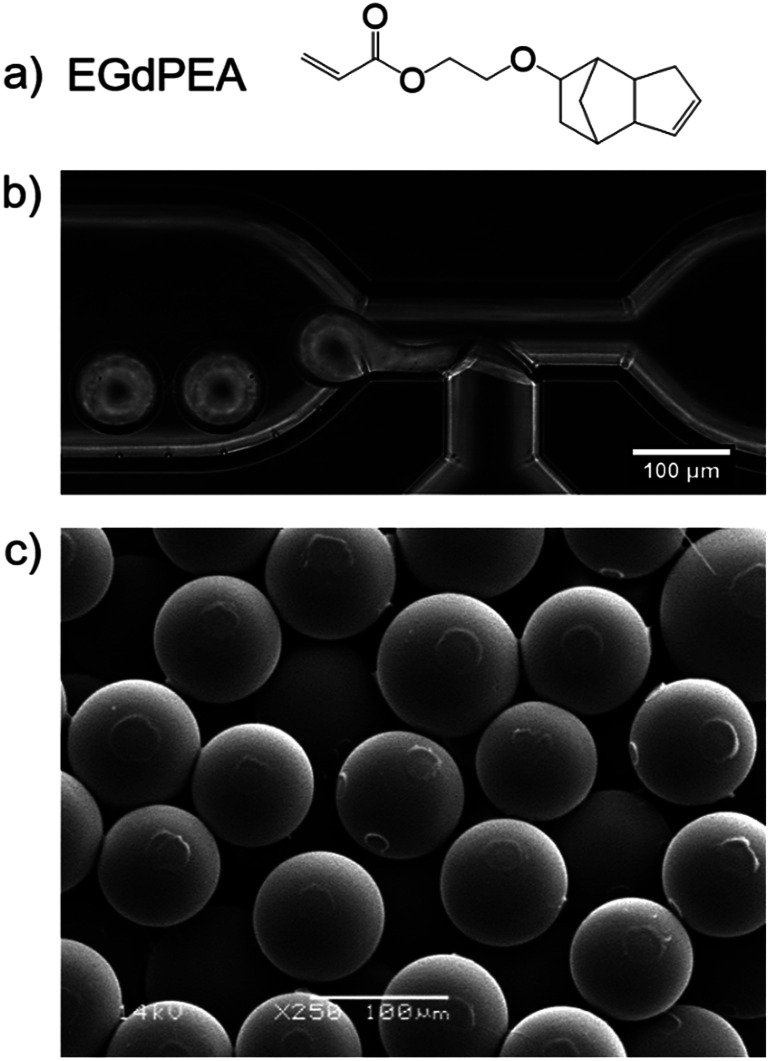
Microparticle formation using microfluidics: (a) chemical structure of the monomer ethylene glycol dicyclopentenyl ether acrylate (EGdPEA), (b) image taken at a glass T-junction: successful formation of monomer droplets using 2 wt% PVA as surfactant. The flow of the aqueous PVA solution is from right to left. (c) SEM micrograph of polyEGdPEA microparticles produced with a continuous and dispersed phase flow rate of *Q*_c_ = 3 ml h^−1^ and *Q*_d_ = 0.3 ml h^−1^, respectively, at a 250× magnification. The particles exhibited an average diameter of 76 ± 1.7 μm. Scale bars: 100 μm.

Microparticles were successfully prepared from the monomer ethylene glycol dicyclopentenyl ether acrylate (EGdPEA) in a microfluidic approach using a glass T-junction chip with a feature size of 100 μm. EGdPEA was selected since it has been identified as a ‘hit’ homopolymer exhibiting low surface area coverage when incubated with *P. aeruginosa*.^2^ The underlying mechanism by which the polyEGdPEA resists biofilm formation has still not been fully elucidated and is part of ongoing research. The chemical structure of the EGdPEA monomer is depicted in Fig. 1(a). The formation of monomer droplets in the glass T-junction is shown in Fig. 1(b). The continuous phase emerging from the right channel is an aqueous poly(vinyl alcohol-*co*-vinyl acetate) (PVA) solution (2 wt%), while the dispersed phase coming from the bottom is the respective monomer solution, in this case comprising of EGdPEA and 1 wt% of 2,2-dimethoxy-2-phenylacetophenone (DMPA) as photo-initiator for the subsequent polymerization of the monomer droplets to microparticles.

PVA is able to stabilize the monomer solution droplets within the deionized water due to its amphiphilicity, hence the surfactant was required for successful droplet formation. Without a surfactant in the continuous phase, a constant stream of monomer solution was observed in the microfluidic channel. Moreover, employing a PVA concentration below 2 wt% failed to produce droplets or led to unstable droplet formation which resulted in a lack of both control and particle homogeneity. No data was gathered for a PVA concentration above 2 wt% because the scope was to reduce the amount of surfactant as much as possible.

Using the methodology outlined, homogeneous microparticles with a coefficient of variation of 2.2% were obtained. Fig. 1(c) shows a micrograph of polyEGdPEA microparticles with an average diameter of 76 μm imaged by SEM. The circular surface features arise from particle to particle contact prior to full hardening in the collection vial.

### Assessment of surface chemistry using ToF-SIMS

**Fig. 2:**
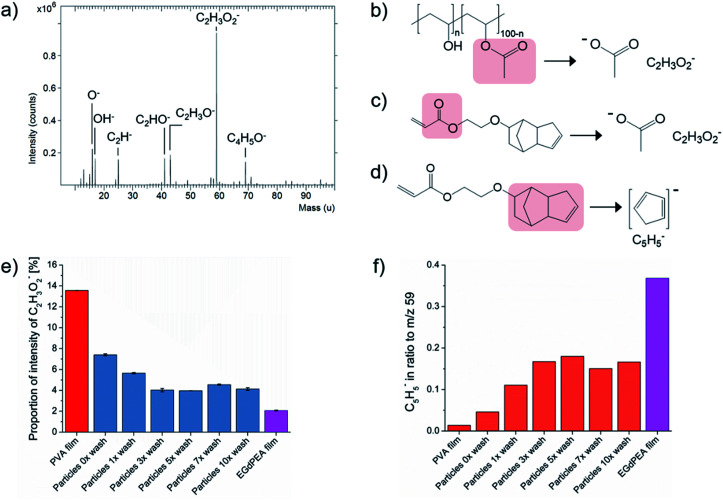
Surface chemistry of polyEGdPEA microparticles and the corresponding flat film: (a) ToF-SIMS spectra of PVA powder as-received acquired in the negative ion polarity, (b) the fragmentation of poly(vinyl alcohol-*co*-vinyl acetate) (PVA) into the acetate anion (CH_3_COO^−^, *m*/*z* = 59) is presented, (c) the fragmentation of the monomer EGdPEA into the secondary ion reveals peaks at the same *m*/*z* positions shown to be representative for the PVA surfactant, (d) the fragmentation of the monomer EGdPEA into the cyclopentadienyl anion (C_5_H_5_^−^), (e) the proportion of total counts [%] of the acetate anion with its standard deviation and (f) the ratio of the cyclopentadienyl anion to the acetate anion (*m*/*z* = 59) for the polyEGdPEA microparticles varying in wash cycles and its corresponding flat thin film. PVA (*M*_w_ 25 000) with a hydrolysis degree of 88% was used as particle surfactant and control.

The surface chemistry of polyEGdPEA particles produced *via* microfluidics was assessed using ToF-SIMS. This surface mass spectral technique is not inherently quantitative, but is good at providing a relative measure of the amount of a particular species in a similar sample down to sub-monolayer sensitivities by virtue of its 3 nm depth of analysis.^26^ To study the presence of PVA surfactant on the polymer particles, aliquots of particles were washed with varying the numbers of wash cycles. A wash cycle involved sonicating the particles in isopropanol for 5 min followed by another sonication in distilled water for 5 min. SIMS spectra of PVA powder as-received (Fig. 2(a)) and a flat thin PVA-free film made of polyEGdPEA were acquired as references for PVA and the particle polymer itself. The secondary ions that are most dominant for detecting PVA arose from the unhydrolyzed vinyl acetate component within the 88% hydrolyzed PVA (structure in Fig. 2(b)) used as particle surfactant and control in this study. The peak detected at *m*/*z* = 59 in the negative SIMS spectrum represents the acetate anion [CH_3_COO]^−^. Fragmentation of the PVA into the acetate anion is illustrated in Fig. 2(b). In the positive ion spectrum, the base peak was found at *m*/*z* = 43 corresponding to the acetyl cation [CH_3_CO]^+^ which had previously been reported by Scholes *et al.* as a diagnostic peak for PVA.^27^ Both peaks, however, cannot only be attributed to ion fragments from the PVA surfactant but also to secondary ions derived from the polymer itself. This was confirmed by the flat polyEGdPEA film revealing peaks at the same *m*/*z* in both secondary ion polarities as shown to be representative for the PVA surfactant (Fig. 2(c)). The proportion of total counts of the acetate anion are plotted for the polyEGdPEA microparticles against the wash cycles and its corresponding flat thin film in Fig. 2(e).

A reduction in intensity of the acetate anion was observed with increased washing as shown in Fig. 2(e). A gradual decrease of 45.7% was quantified from particles as-produced (particles 0× wash) to particles analyzed after the third wash cycle. The normalized counts (%) of the PVA characteristic secondary ion remained reasonably constant without any significant changes from the 3rd to the 10th wash cycle. Increased values, however, were obtained on microparticles after the 7th wash. One of the possible reasons is that particles, which were initially agglomerated during washing, became separated in the sample preparation for the ToF-SIMS analysis. Consequently, not all particles were washed with the same efficiency leaving a higher amount of PVA surfactant behind. The same observations were made in the positive ion SIMS spectra when considering the acetyl cation.

A secondary ion representative of the EGdPEA polymer, C_5_H_5_^−^, was selected to verify the increase in the polymer concentration with decreased amount of PVA surfactant at the surfaces. The fragmentation of the EGdPEA into the cyclopentadienyl anion (C_5_H_5_^−^) is presented in Fig. 2(d). An increase in the ratio of the cyclopentadienyl anion to the acetate anion of 264.2% was observed from particles as-produced (particles 0× wash) to particles after the 3rd wash cycle (Fig. 2(f)). The increase of the anion intensity characteristic for the EGdPEA fragment was inversely proportional to the decrease in intensity found for the PVA compound, confirming that the PVA surfactant was removed from the particle surfaces. However, the intensity of the cyclopentadienyl anion after extensive washing did not reach the same intensity that was observed in a prepared film known to be high purity EGdPEA, suggesting that not all the PVA was removed by washing. Consequently, it was hypothesized that most of the PVA surfactant was physically entangled in the polymeric particle surfaces preventing its complete removal.

**Fig. 3:**
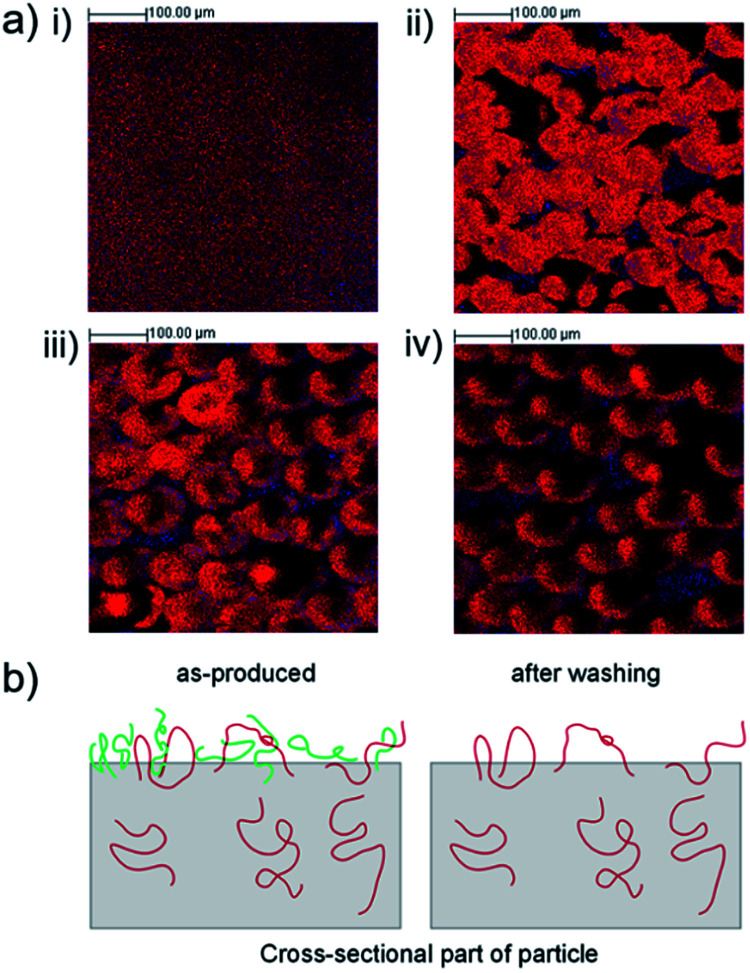
Distribution of PVA surfactant and EGdPEA on surfaces of microfluidic produced polyEGdPEA particles and the corresponding flat film: (a) negative ToF-SIMS imaging showing the overlay of CH_3_COO^−^ (red, PVA) and C_5_H_5_^−^ (blue, EGdPEA) for flat film (i), untreated microspheres (particles as-produced) (ii), particles after performing one (iii) and 10 (iv) wash cycles. The ToF-SIMS image acquired after the first wash (iii) reveals an inhomogeneous distribution of PVA surfactant on microparticles. The images became distorted in the SIMS imaging process as a result of the particle shape. (b) Schematic representation of the cross-sectional part of a particle showing physically entangled PVA chains (red) and non-entangled PVA chains (green) which are removed by extensive washing.

Applying the characteristic ions, C_2_H_3_O_2_^−^ and C_5_H_5_^−^, the ToF-SIMS spectra were reconstructed as images to examine the distribution of PVA surfactant and EGdPEA on the surfaces. The overlay of PVA surfactant (red) and EGdPEA (blue) is shown for the thin film (i), particles as-produced (ii), after the first wash (iii) and after 10 washes (iv) in Fig. 3(a). The particle shape became distorted in the SIMS imaging process as a result of the particle shape and primary ion beam configuration.^28^ The flat thin film revealed a homogeneous distribution of the two diagnostic ions across the surface. In contrast, the untreated EGdPEA particles (no washing) were almost fully covered by the PVA surfactant such that the proportion of the characteristic ion C_5_H_5_^−^ to the PVA was only 0.05. PolyEGdPEA appeared only on a few spots in the image (Fig. 3(a)(ii)) indicating that the PVA surfactant might cover the surfaces as a discontinuous layer as previously reported by Rafati *et al.*^19^ These images, however, cannot be resolved in such a detail to confirm this possibility. Comparing the ToF-SIMS images of the microparticles as-produced (ii) with the image of the extensively washed particles (iv), a significant reduction in PVA was observed. Therefore, the reduction in PVA surfactant at the particle surfaces resulted in an increased detection of the cyclopentadienyl anion derived from the EGdPEA polymer (Fig. S4(b)†).

The distribution of PVA surfactant was further analyzed on microparticles after the first wash because microfluidic produced particles were washed once with phosphate buffer saline (PBS) before being tested for bacterial attachment. As shown in Fig. 3(a)(iii), the concentration of PVA surfactant was not uniform within a sample aliquot.

A model proposing the PVA chain entanglement is illustrated in Fig. 3(b). Microparticles as-produced reveal both polymeric PVA chains physically entangled (red) and loosely bound (green) to the particle surface. Washing the particles excessively leads to removal of the loosely attached PVA chains leaving the physically entangled PVA chains behind.

### Effect of PVA on bacterial attachment on washed microparticles

**Fig. 4:**
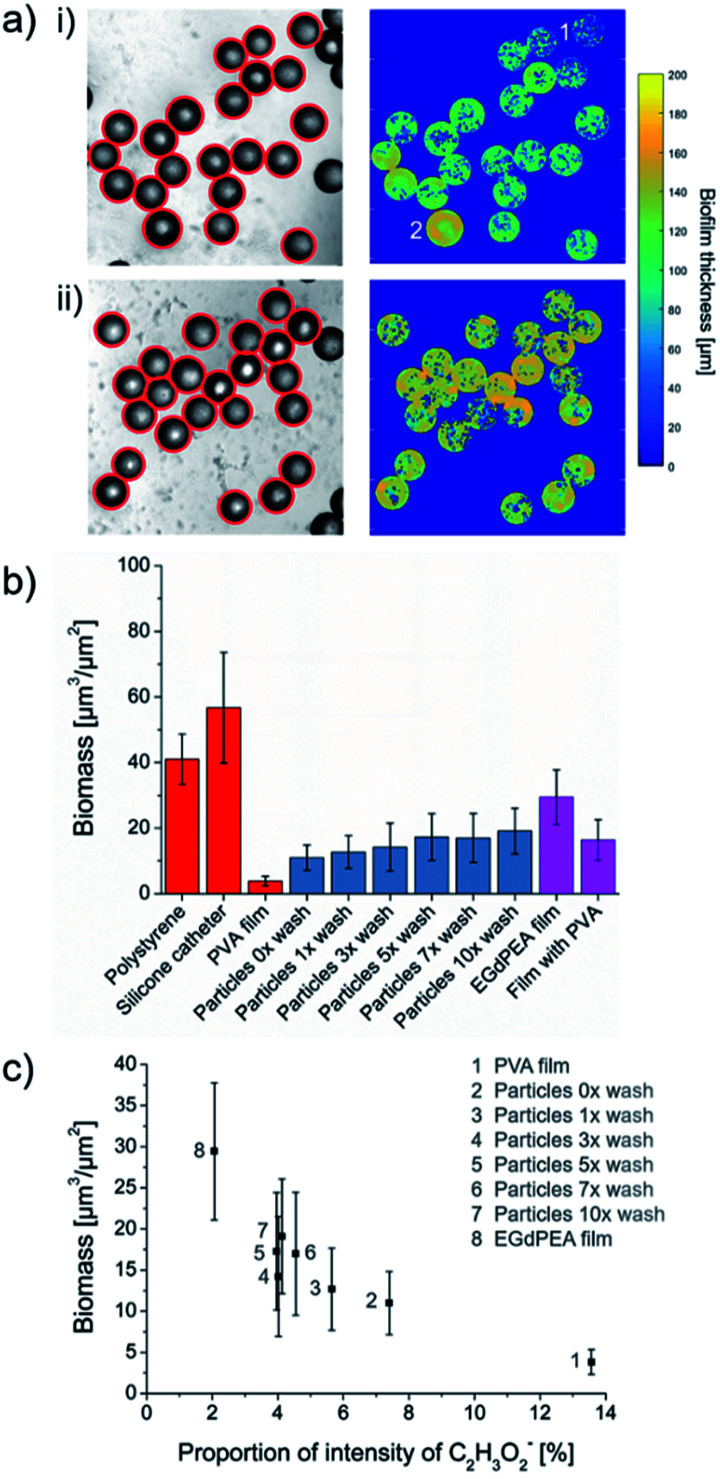
Effect of PVA surfactant on bacterial attachment on microfluidic produced polyEGdPEA particles and flat films. (a) Bacterial cells attached on polyEGdPEA microparticles showing the successful circle detection on acquired brightfield images (left) and the determined biomass (right): (i) particles as-produced (no washing), (ii) particles washed 10 times. Quantification of biomass on polyEGdPEA particles varying in repeats of wash cycles and pipetted polyEGdPEA films: (b) biomass is plotted against the samples tested, whereas non-tissue culture polystyrene and pieces of silicone catheter served as references for high bacterial attachment surfaces, (c) correlation between biomass captured on surfaces and the proportion of total counts of the acetate anion [CH_3_COO]^−^ detected using ToF-SIMS.

To study the effect of residual PVA surfactant on the ability of the particle to resist bacterial attachment, 5 mg each of microparticle sample varying in the number of wash cycles were cultured with *P. aeruginosa* PAO1-N expressing the mCherry fluorescent protein for 24 h in 48-well microtitre plates with agitation (shaking at 60 rpm) and imaged using confocal microscopy. The number of bacterial cells attached was quantified using the mCherry fluorescence, exclusively on the surfaces of microparticles and flat films by using a custom MATLAB algorithm (details in ESI 1.7†). Thin films, made by pipetting the EGdPEA monomer into microtitre well plates, were photopolymerized in the presence of a 2 wt% aqueous PVA solution. Curing the acrylate in the presence of PVA was conducted to mimic the microfluidic process of crosslinking PVA surfactant into particle surfaces as confirmed by ToF-SIMS analysis. The bacterial attachment quantification method allowed assessment of the variance of biomass within a sample. The successful particle detection on optical brightfield images and the captured biomass, resulting from applying the mask throughout the volumetric images, is depicted in Fig. 4(a) for microfluidic produced polyEGdPEA particles as-produced (i) and after 10 wash cycles (ii); wash cycles were performed before incubation in medium inoculated with PAO1-N mCherry. The biofilm thickness detected across the particle surfaces varied which is best seen in Fig. 4(a)(i). The particle numbered 2 exhibited considerably more bacterial cells attached compared to the particle numbered 1. These differences in biomass volume may have developed due to variations in PVA surface coverage (Fig. 3(a)) or due to heterogeneity of colonization across microparticles within a test aliquot. Moreover, alterations in surface topography were proposed by Feng *et al.*^29^ to lead to different levels of bacterial attachment. This study revealed that bacterial attachment was reduced on anodic alumina surfaces with cylindrical nanopores with diameters of 15 and 25 nm as compared with surfaces with larger pores. The bacteria-repelling effect of the topographies was observed across several bacterial species and attributed to densely distributed pores exerting increased repulsive forces on the bacteria found in the proximity of the surfaces.^29^ In contrast to Feng *et al.*, the microfluidic produced polyEGdPEA particles exhibited mostly detachment marks and only few surface pores on some particles, as shown in the Fig. S5.†

The overall biomass for each sample is shown in Fig. 4(b) including comparison samples of high bacterial attachment surfaces: non-tissue culture polystyrene (bottom of well) and sections of a silicone catheter. A significantly lower bacterial attachment (90.7% reduction) was quantified on PVA compared with polystyrene. The PVA sample consisted of 5 mg of loose PVA powder in a microtitre plate well that dissolved during sample washing and testing. This observation revealed that the low-fouling properties of the well surfaces covered with dissolved bulk PVA were similar to that reported by Salman *et al.* on PVA films (5 wt%) cast on glass and plastic plates.^21^ For the microfluidic produced polyEGdPEA microparticles, a gradual increase in the bacterial cells attached was observed from zero up to three particle pre-wash cycles. However, the level of bacterial attachment remained unaltered after performing five wash cycles. The number of bacterial cells attached was inversely proportional to the amount of PVA surfactant determined on the particle surfaces using ToF-SIMS (shown in Fig. 2(c)). Consequently, the reduction in PA01-N mCherry attachment observed with higher PVA surfactant concentration at the particle surfaces confirmed an anti-attachment effect of the surfactant. Furthermore, these findings were substantiated when considering the reduction in bacterial attachment of 44.6% on the PVA embedded polyEGdPEA films compared to the control films without PVA.

The relationship between bacterial attachment and PVA concentration is shown by plotting the biomass captured on PVA, polyEGdPEA particles and films against the proportion of total counts of the ToF-SIMS acetate ion representative for PVA (Fig. 4(c)). The graph demonstrates that having more PVA on the surface resulted in less biomass. The number of bacterial cells quantified on polyEGdPEA particles and films was higher than the amount determined on bulk PVA. This could be explained by two possible reasons: first, differences in the low-attachment mechanism for PVA bulk samples and PVA surfactant physically entrapped into polymerized surfaces; second, differences in the surface area of PVA exposed to bacterial cells. It was postulated that PVA in the bulk works by dissolution of the PVA followed by sloughing off the bacterial attachment from the weak interfacial layer. However, in the case of the rinsed samples exhibiting PVA surfactant which cannot be fully removed from the particle surfaces, this proposed mechanism of action for the bulk PVA would not be applicable. PVA physically entangled into the surface (surfactant) is considered as a water-soluble polymer that can induce surface hydration *via* hydrogen bonding. A tightly bound layer of water molecules near the surface is known to form a physical and energetic barrier able to prevent stable bacterial attachment.^30^ It is believed that PVA coatings to prevent bacterial attachment would be a short-lived strategy since it is likely to be depleted over time.

## Conclusions

In this study, polyEGdPEA microparticles were successfully produced with average sizes of 76 ± 1.7 μm by combining microfluidics with photopolymerization of an acrylate monomer. It was demonstrated that surfactant was needed to achieve stable droplet formation, in this work 2 wt% aqueous PVA solution. ToF-SIMS analysis revealed the presence of PVA surfactant on the particle surfaces, which was partially removed through excessive washing (45.7% reduction). A chain entanglement model was proposed to explain the residual PVA. A bulk PVA sample had 91% less *P. aeruginosa* cell coverage compared to polystyrene. By testing microparticles varying in repeats of wash cycles, a trend was observed which revealed greater reduction in bacterial attachment for particles with increased amount of retained PVA surfactant at particle surfaces. The finding of this work emphasizes the role of surfactants in controlling cellular response to materials in this system, and the importance of carrying out chemical surface analysis to detect and rationalize the role of such confounding species. This insight will help improve the understanding of the structure–function relationships and hence, facilitate to move towards rational materials design with biofilm control in the future.

## Author contributions

The manuscript was written through contributions of all authors. All authors have given approval to the final version of the manuscript.

## Conflicts of interest

The authors declare no competing financial interest.

## Supplementary Material

RA-008-C8RA01491D-s001

RA-008-C8RA01491D-s002

RA-008-C8RA01491D-s003

## References

[cit1] Celiz A. D., Smith J. G. W., Patel A. K., Langer R., Anderson D. G., Barrett D. A., Young L. E., Davies M. C., Denning C., Alexander M. R. (2014). Biomater. Sci..

[cit2] Hook A. L., Chang C.-Y., Yang J., Luckett J., Cockayne A., Atkinson S., Mei Y., Bayston R., Irvine D. J., Langer R., Anderson D. G., Williams P., Davies M. C., Alexander M. R. (2012). Nat. Biotechnol..

[cit3] Brocchini S., James K., Tangpasuthadol V., Kohn J. (1998). J. Biomed. Mater. Res..

[cit4] Davies M. C., Alexander M. R., Hook A. L., Yang J., Mei Y., Taylor M., Urquhart A. J., Langer R., Anderson D. G. (2010). J. Drug Targeting.

[cit5] Curtis A., Wilkinson C. (1997). Biomaterials.

[cit6] Iwanaga S., Saito N., Sanae H., Nakamura M. (2013). Colloids Surf., B.

[cit7] Siegwart D. J., Whitehead K. a, Nuhn L., Sahay G., Cheng H., Jiang S., Ma M., Lytton-Jean A., Vegas A., Fenton P., Levins C. G., Love K. T., Lee H., Cortez C., Collins S. P., Li Y. F., Jang J., Querbes W., Zurenko C., Novobrantseva T., Langer R., Anderson D. G. (2011). Proc. Natl. Acad. Sci. U. S. A..

[cit8] Ma M., Chiu A., Sahay G., Doloff J. C., Dholakia N., Thakrar R., Cohen J., Vegas A., Chen D., Bratlie K. M., Dang T., York R. L., Hollister-Lock J., Weir G. C., Anderson D. G. (2013). Adv. Healthcare Mater..

[cit9] Chau D. Y. S., Agashi K., Shakesheff K. M. (2008). Mater. Sci. Technol..

[cit10] Ferrara K., Pollard R., Borden M. (2007). Annu. Rev. Biomed. Eng..

[cit11] Dendukuri D., Doyle P. S. (2009). Adv. Mater..

[cit12] Li G. L., Möhwald H., Shchukin D. G. (2013). Chem. Soc. Rev..

[cit13] Sinha V. R., Trehan A. (2003). J. Controlled Release.

[cit14] Kim J. H., Jeon T. Y., Choi T. M., Shim T. S., Kim S.-H., Yang S.-M. (2014). Langmuir.

[cit15] Nisisako T., Hatsuzawa T. (2010). Microfluid. Nanofluid..

[cit16] Baah D., Floyd-Smith T. (2014). Microfluid. Nanofluid..

[cit17] Vasiliauskas R., Liu D., Cito S., Zhang H., Shahbazi M.-A., Sikanen T., Mazutis L., Santos H. a. (2015). ACS Appl. Mater. Interfaces.

[cit18] Nisisako T., Torii T. (2008). Lab Chip.

[cit19] Rafati A., Boussahel A., Shakesheff K. M., Shard A. G., Roberts C. J., Chen X., Scurr D. J., Rigby-singleton S., Whiteside P., Alexander M. R., Davies M. C. (2012). J. Controlled Release.

[cit20] Anna S. L., Bontoux N., Stone H. A. (2003). Appl. Phys. Lett..

[cit21] Salman J. A. S., Kadhemy M. F. H., Jaleel M. S., Abdal A. K. (2014). Int. J. Curr. Microbiol. Appl. Sci..

[cit22] J. O'Neill, Comm. by UK Prime Minist

[cit23] Callow M. E., Callow J. A. (2002). Biologist.

[cit24] Cao S., Wang J., Chen H., Chen D. (2011). Chin. Sci. Bull..

[cit25] Monds R. D., O'Toole G. A. (2009). Trends Microbiol..

[cit26] Ogaki R., Green F. M., Gilmore I. S., Shard A. G., Luk S., Alexander M. R., Davies M. C. (2007). Surf. Interface Anal..

[cit27] Scholes P. D., Coombes A. G. A., Illum L., Davis S. S., Watts J. F., Ustariz C., Vert M., Davies M. C. (1999). J. Controlled Release.

[cit28] Vickerman J. C. and Briggs D., TOF-SIMS: Materials Analysis by Mass Spectrometry, IM Publications, 2nd edn, 2013

[cit29] Feng G., Cheng Y., Wang S.-Y., Borca-Tasciuc D. A., Worobo R. W., Moraru C. I. (2015). NPJ Biofilms Microbiomes.

[cit30] Chen S., Li L., Zhao C., Zheng J. (2010). Polymer (Guildf).

