# Correction: Effect of surfactant on *Pseudomonas aeruginosa* colonization of polymer microparticles and flat films

**DOI:** 10.1039/c8ra90042f

**Published:** 2018-05-24

**Authors:** Amanda Hüsler, Simon Haas, Luke Parry, Manuel Romero, Takasi Nisisako, Paul Williams, Ricky D. Wildman, Morgan R. Alexander

**Affiliations:** Advanced Materials and Healthcare Technologies Division, School of Pharmacy, University of Nottingham Nottingham NG7 2RD UK morgan.alexander@nottingham.ac.uk; Centre for Additive Manufacturing, Faculty of Engineering, University of Nottingham Nottingham NG7 2RD UK; Institute of Innovative Research, Tokyo Institute of Technology Yokohama Japan; Centre for Biomolecular Sciences, School of Life Sciences, University of Nottingham Nottingham NG7 2RD UK

## Abstract

Correction for ‘Effect of surfactant on *Pseudomonas aeruginosa* colonization of polymer microparticles and flat films’ by Amanda Hüsler *et al.*, *RSC Adv.*, 2018, **8**, 15352–15357.

The authors regret that the following information was not included in the original manuscript. All relevant data are available from the University of Nottingham’s Research Data Management Repository under the DOI: 10.17639/nott.352, available at the following address: http://dx.doi.org/10.17639/nott.352

The Royal Society of Chemistry apologises for these errors and any consequent inconvenience to authors and readers.

## Supplementary Material

