# Supplementary material for: Effect of surfactant on Pseudomonas aeruginosa colonization of polymer microparticles and flat films
Source: RSC Adv. 2018 Apr 24;8(28):15352–7. doi: 10.1039/c8ra01491d (PMC9079973; doi:10.1039/c8ra01491d)
Supplement: RA-008-C8RA01491D-s001 [file RA-008-C8RA01491D-s001.pdf]

Electronic Supplementary Material (ESI) for RSC Advances.
This journal is © The Royal Society of Chemistry 2018
Please do not adjust margins

RSC Advances

Supplementary Information

Effect of surfactant on *Pseudomonas aeruginosa* colonization of polymer microparticles and flat films†

Received 00th January 20xx,
Accepted 00th January 20xx

DOI: 10.1039/x0xx00000x

www.rsc.org/

Amanda Hüsler[a,b,c], Simon Haas[b], Luke Parry[b], Manuel Romero[d], Takasi Nisisako[c], Paul Williams[d], Ricky D. Wildman[b], Morgan R. Alexander*[a]

Experimental Details

Preparation of polymer microparticles

Acrylate based microparticles were produced in a microfluidic approach using a simple T-junction glass chip (part number: 3000158, Dolomite Microfluidics, UK). The continuous (Qc) and dispersed (Qd) phase were pushed through the channels at a constant speed (Qc = 3 ml/h, Qd = 0.3 ml/h) using PHD Ultra syringe pumps (Biochrom Ltd, UK), Hamilton series 1000 glass syringes (Sigma-Aldrich Company Ltd, UK) and 1/16" OD, 0.25 mm ID FEP tubing (Dolomite Microfluidics, UK). For the continuous phase, poly(vinyl alcohol-co-vinyl acetate) (PVA) (Mw 25,000, 88% hydrolyzed, Fischer Scientific UK Ltd, UK) was dissolved in deionized water to prepare a 2 wt% aqueous PVA solution. The dispersed phase was composed of the ethylene glycol dicyclopentenyl ether acrylate (EGdPEA) (Sigma-Aldrich Company Ltd, UK) monomer with 1 wt% photoinitiator 2,2-dimethoxy-2-phenylacetophenone (DMPA) (Sigma-Aldrich Company Ltd, UK). After droplet formation, the monomer solution was photopolymerized with a LX 180 LED spot curing source (wavelength of 365 nm, 8.7 W/cm^2, Jenton International, UK). Droplet formation was followed with a Ti-S inverted fluorescence microscope (Nikon UK Ltd, UK) at a magnification of 20x with a NA of 0.45 and a working distance of WD = 6.9-8.2 mm. Videos and images of the process were recorded with a UX100 Mini high-speed camera (Photron Ltd, UK) at a frame rate of 10,000– 20,000 frames per second, a resolution of 1240 × 480 pixels and a pixel size of 10 µm. The particles were collected in a vial containing 2 wt% aqueous PVA solution. Afterwards, the particles were filtered through a nylon mesh with 40 µm pore size (Plastok Ltd, UK) and dried at room temperature.

[a] Advanced Materials and Healthcare Technologies Division, School of Pharmacy, University of Nottingham, Nottingham, NG7 2RD.
[b] Centre for Additive Manufacturing, Faculty of Engineering, University of Nottingham, Nottingham, NG7 2RD.
[c] Institute of Innovative Research, Tokyo Institute of Technology, Yokohama, Japan.
[d] Centre for Biomolecular Sciences, School of Life Sciences, University of Nottingham, Nottingham, NG7 2RD.

Flat thin film formation

Control films were prepared by pipetting 40 µl of a monomer solution onto the bottom of a well in a Falcon® non-tissue culture 48-well plate (Corning Inc., UK). The resultant computed film thickness was 421 µm. For subsequent curing, the plate was exposed to UV light (Blak-Ray® XX-15L UV Bench Lamp, 365 nm) for 30 min under an argon atmosphere. Monomer solutions consisted of acrylate based monomers with 1 wt% DMPA. A reduced oxygen atmosphere (argon) was necessary to produce flat thin films since oxygen acts as an inhibitor for the polymerisation by quenching free radicals.
Polymeric films with PVA surfactant molecules physically entangled into the surface, referred to as 'PVA embedded films', were fabricated by placing 1 ml of a 2 wt% aqueous PVA solution on top of the pipetted monomer solutions. Immediately thereafter, the films in the well plate were irradiated with a UV source for 30 min under an argon atmosphere. After the polymerization, residues of the PVA solution were removed from the wells using a pipette. A schematic drawing of the preparation of flat control and PVA embedded films is illustrated in Fig. S1.

Scanning electron microscopy

SEM imaging was conducted with a JEOL JSM-6060LV scanning electron microscope (JEOL Ltd., Japan) at 10 – 20 kV to investigate the shape and surface morphology of the polymeric microspheres. The microfluidic produced particles were deposited, using a spatula, onto a double-sided adhesive carbon tape. Prior to SEM analysis, the samples were gold-coated for 4-5 minutes at 25 mA in an argon atmosphere using a Leica EM SCD005 sputter coater (Leica Microsystems GmbH, Germany) to yield a ~25 nm thick coating.

Time-of-Flight Secondary Ion Mass Spectrometry

ToF-SIMS measurements were performed using an IONTOF IV ToF-SIMS instrument (ION-TOF GmbH, Germany) operated

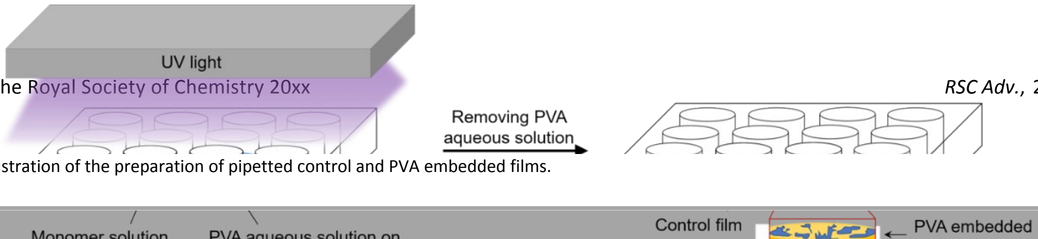

Fig. S1: Schematic illustration of the preparation of pipetted control and PVA embedded films.

Please do not adjust margins

using a 25 kV Bi^{3+} primary ion source in 'high current bunched' mode. The primary ions exhibited a pulsed target current of ~1 pA. Sample areas of 500 × 500 µm were acquired at a resolution of 256 × 256 pixels by rastering the primary ion beam in a "random path" over the substrate surface. An ion dose of 2.45 × 1011 ions per cm^2 was applied to each sample area, ensuring static conditions were maintained throughout. Both positive and negative secondary ion spectra were collected (mass resolution of >7000) over an acquisition period of 20 scans (the data from which was summed). Due to the non-conductive nature of the polymeric samples, charge compensation was applied in the form of a low energy (20 eV) electron flood gun.

Bacterial attachment studies

The effect of PVA surfactant on bacterial attachment and subsequent biofilm formation was investigated using microfluidic produced microparticles and flat films. Red fluorescence emitting *Pseudomonas aeruginosa* (*P. aeruginosa*) PAO1-N expressing the fluorescent mCherry protein was routinely plated out on LB (Luria-Bertani, UK) agar plates and grown overnight at 37°C. The following day, a single colony was selected and added to a Falcon™ tube filled with 10 ml LB medium to produce a primary culture. This tube was then incubated at 37°C with shaking at 200 rpm. The next day, the bacteria were pelleted by centrifugation at 9500 rpm for 5 min and the supernatant was removed. The bacteria were re-suspended in RPMI-1640 medium (R0883, Sigma, UK) and pelleted again to remove LB medium residues. Prior to incubation with the bacteria, the films and microparticles in non-tissue culture 48-well plates were washed with phosphate buffer saline (PBS), air-dried and UV sterilized for 30 min using a desktop UV sterilization unit. The samples were incubated each in 1 ml RPMI-1640 inoculated with diluted (OD_{600} = 0.01) PAO1-N mCherry from overnight cultures. The incubation was performed at 37°C with shaking at 60 rpm for 24h. After incubating the 48-well plate for 24 h, the samples were washed with sterile PBS (3 × 600 µl) at room temperature prior to analysis. The washing steps were conducted as carefully as possible using a pipette to remove non-adhesive bacteria but not to damage the biofilms.

Confocal microscope image acquisition

The bacterial attachment coverage on microparticles and flat thin films was observed using a ZEISS LSM 700 laser confocal microscope. Fluorescence images were acquired representing an area of 639 × 639 µm with a resolution of 512 × 512 pixels at 8-bit color depth. Image acquisition was conducted with 2 light channels, mCherry and T-PMT, and consisted of optical cross-sections which were evenly spaced by a vertical step of 4.0 and 1.5 µm for films and microfluidic produced particles, respectively. Stacking these optical cross-sections acquired at different depths within a sample, a 3D image can be reconstructed. The microscope was operated as follows: excitation at 555 nm with a power of 10%, 10x/0.30 M27 EC Plan-Neofluar objective at an Airy disc setting of 1 Airy unit (AU) which corresponds to 34 µm.

Confocal image processing

The amount of bacteria observed on the flat films and microparticles was calculated using Matlab (R2016b). The volumetric fluorescence images were directly imported into Matlab and the in-built 3D median filter (*medfilt3*) was applied to filter noise present in the confocal data. Automatic thresholding and segmentation of volumes were statistically determined for the confocal microscopy images using the objective threshold selection (OTS) procedure proposed by Xavier et al.[1]. This provides a consistent method selecting the threshold value without requiring manual intervention.
To quantify the level of bacterial attachment on the surface of microparticles, the particle centers and radii were automatically found by detecting circles in the optical brightfield images using the built-in Matlab circle detection function (*imfindcircles*), as shown in Fig. S2 a). Using these geometries, a cylindrical mask was projected vertically and applied to the volumetric fluorescence images to capture only the biomass present on the selection of detected particles (Fig. S2 b)).

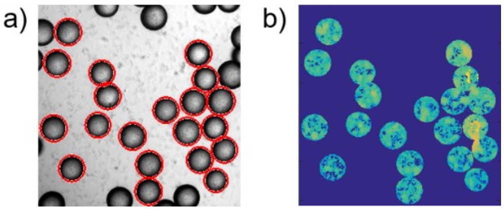

Fig. S2: Biomass quantification on microparticles: a) successful circle detection obtained from the optical image of microparticles, b) biomass captured on particles after applying the cylindrical mask to the volumetric image.

The bacterial attachment was calculated analogous to COMSTAT, a software that facilitate quantification of three-dimensional biofilm structures[2]. Due to the lack of reports about quantification of biofilm-associated bacteria on 3D surfaces (here, particles) in the literature, a new algorithm was developed for this work. The overall biovolume on microspheres was determined by summing the thresholded fluorescent biomass pixels in all images of a stack multiplied by the voxel size $[pixel\ size_x \times pixel\ size_y \times pixel\ size_z]$ and dividing by the total spherical surface area of the particles detected. The resulting biomass volume per substratum area constitutes the overall volume of the bacteria attached and also provides an estimate of the biomass in the biofilm[2]. Similarly, the biovolume of the flat films was calculated by summing the threshold volumes and dividing by the planar area of the image. The biofilm thickness is defined as the maximum thickness over a given location by locating the highest point (µm) above each (x, y) pixel in the bottom layer containing biomass[2]. This calculation was automatically repeated throughout all samples relevant for this study, with the particle detection validated visually.

This journal is © The Royal Society of Chemistry 20xx

Please do not adjust margins

Please do not adjust margins

Additional Results

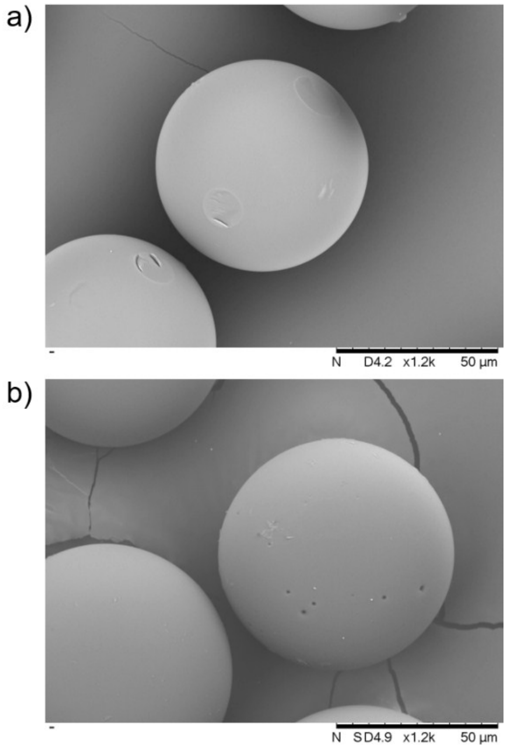

Fig. S3: Microfluidic produced polyEGdPEA microparticles tested with PA01-N mCherry and analyzed as-produced using SEM. SEM micrographs revealing two different defects detected on the surfaces of polyEGdPEA microparticles: a) detachment marks, b) surface porosity.

Effect of PVA on bacterial attachment on washed microparticles

References

1 J. B. Xavier, A. Schnell, S. Wuertz, R. Palmer, D. C. White and J. S. Almeida, *J. Microbiol. Methods*, 2001, **47**, 169–180.

2 A. Heydorn, A. Heydorn, A. T. Nielsen, A. T. Nielsen, M. Hentzer and M. Hentzer, *Microbiology*, 2000, **146**, 2395–2407.

Please do not adjust margins
